# Study of Upper Pharyngeal Airway Dimension in Young Adults Visiting Orthodontic Department of a Dental College: A Descriptive Crosssectional Study

**DOI:** 10.31729/jnma.6293

**Published:** 2021-03-31

**Authors:** Resina Pradhan, Anjana Rajbhandari, Manju Bajracharya, Pushkar Manandhar, Surendra Maharjan, Bashu Dev Pant

**Affiliations:** 1 Department of Orthodontics, People's Dental College and Hospital, Kathmandu, Nepal

**Keywords:** *airway*, *hyoid bone position*, *tongue dimension*

## Abstract

**Introduction:**

Orthodontic treatment may affect the size and position of oropharyngeal structures altering the airway dimension. Airway dimension is considered as one of the parameter of orthodontic diagnosis and treatment planning. Narrow airway passage is associated with sleep disordered breathing. This aims to study about the measurement of upper pharyngeal airway dimension of young Nepalese adults visiting orthodontic department of a dental college.

**Methods:**

This descriptive cross-sectional study conducted at Department of Orthodontics, People's Dental College and Hospital, Kathmandu. Data was collected from 8^th^ December 2020 to 28^th^ February 2021. Ethical clearance (Reference number. 1, CH100 06, 2077/2076) was taken from Institutional Review Committee of Peoples Dental College and Hospital, Kathmandu, Nepal. Convenient Sampling technique was done. Data was collected and analysis was done using Statistical Package of Social Sciences 16.

**Results:**

Mean upper pharyngeal airway dimension was 11.40±1.84mm and 11.68±1.96mm for male and female respectively. Upper pharyngeal airway dimension was found to be almost similar for male and female.

**Conclusions:**

Gender diversity regarding upper pharyngeal airway dimesnion was not observed in this study while other studies also revealed similarities in upper pharyngeal airway dimension between male and female, inspite of larger craniofacial dimension in males than females.

## INTRODUCTION

Positional changes of mandible during orthodontic treatment may alter tongue and hyoid bone position affecting pharyngeal airway dimension.^1^ Reduced airway passage is detrimental and has been associated with abnormal growth of craniofacial structures and may be associated with sleep disordered breathing.^2^

Widening the narrow airway passage with mandibular advancement allows anterior positioning of hyoid bone and provides normal breathing and normal growth of craniofacial structures.^3^ There has been various studies to measure upper pharyngeal airway in individual with normal craniofacial structures as well as abnormal facial pattern. ^4^

As stated in literatures airway dimension, tongue size and hyoid bone position are highly reproducible in lateral cephalogram.^5^ The objective of this study is to measure upper pharyngeal airway dimension in young Nepalese adults visting orthodontic department of a dental college of Kathmandu.

## METHODS

The study design was descriptive cross-sectional study conducted at Department of Orthodontics, Peoples Dental College and Hospital, Kathmandu from 8^th^ December 2020 to 28^th^ February 2021. Ethical clearance (Ref. no. 1, CH100 06, 2077/2076) was taken from Institutional Review Committee of People's Dental College and Hospital, Kathmandu, Nepal. Lateral cephalograms of Nepalese young adults aged 18-27 years visiting orthodontic department, seeking orthodontic treatment were selected based on inclusion and exclusion criterion (Angles class I molar and canine relation with normal overjet and overbite, skeletal class I with normal growth pattern were included and mouth breathing habit, craniofacial abnormalities and orthodontic treatment were excluded). Written and signed consent was taken prior to pre-treatment record collection. Pilot study was performed in thirty lateral cephalograms to know standard deviation and error. Then with the adjusted error, sample size was determined to be 114.

$$\begin{array}{ll} \text{n} & =({\text{z}}^{\text{2}}\times {\text{σ}}^{\text{2}})/{\text{e}}^{\text{2}}\\ & =({\left(1.96\right)}^{2}\times {\left(0.6\right)}^{2})/{\left(0.11\right)}^{\text{2}}\\ & =114 \end{array}$$

Where,

Z = 1.96 at 95 % Confidence Interval
*σ* = 0.6 from pilot study
e = margin of error, 0.11

The calculated sample size was 114. Increase in 10% sample size (114 + 11 = 125) was to mitigate error that may arise due to difficulty in land mark localisation.

Lateral cephalograms was obtained using Sirona Orthophos SL. exposed at 73 KV - 15 mA to 84 KV - 13 mA for all patients, with patient in natural head position for reproducibility of landmarks, maximum intercuspation and light lip contact. The patients were asked not to swallow or move tongue or head during the exposure.

For pre-testing thirty lateral cephalograms, which were not included in the study sample, were collected and study variables were analysed prior to the research study. In the selected upper pharyngeal width 125 samples name and sex in the lateral cephalograms were blinded. Using the tracing paper, view box and micro lead 3H pencils fifteen randomly selected lateral cephalograms were traced and landmarks were localized. These lateral cephalograms were relocalised by other investigators to check accuracy in landmark localization. This was followed by tracing, landmark localization and measurement of the parameters of the research study manually by the principal investigator. Twenty five lateral cephalograms was drawn randomly and the parameters were remeasured in one week interval to test intraobserver variation in landmark measurement. All the collected information was recorded in proforma.

As a research parameter, upper pharyngeal airway dimesnsion was measured from a point on anterior half of posterior outline of the soft palate to the closest point on the Posterior Pharyngeal Wall. Lower Pharyngeal Width was measured from the point of intersection of the posterior border of the tongue and the inferior border of the mandible to the closest point on the Posterior Pharyngeal Wall. Hyoid Bone position measured as perpendicular distance from Superior Anterior Point in Hyoid Bone to Mandibular Plane. Tongue Length from base of Epiglottis to Tip of the Tongue. Tongue Height was measured as perpendicular distance from maximum height of dorsum of tongue to Tongue Length.^1^ Measuring scale had 0.5 mm of accuracy for precision in measurement.

Data was fed in Microsoft Excel. Descriptive statistics was drawn using SPSS 16 (Statistical Package of Social Sciences). Frequency distribution of the variables checked in histogram for normal distribution.

Error in measurement was evaluated finding Dahlberg error (1940) to know the impact of the error on final result.

$$D=\sqrt{\sum_{i=1}^{N}\;\frac{d_{i}^{2}}{2N}}$$

Where d_1_ is the difference between the first and second measure, N is the remeasured sample size 25. Dahlberg error for different parameters lied between 0.1 to 0.5 mm with minimum deviation for hyoid bone position and largest deviation for tongue length.^6^ Systematic error in this study has been reduced by using lateral cephalogram from same X-ray machine by single observer. To minimize the systemic error, the data presented in the study is the original data without adjustment for the radiographic magnification of 13%.

## RESULTS

**Figure 1.**
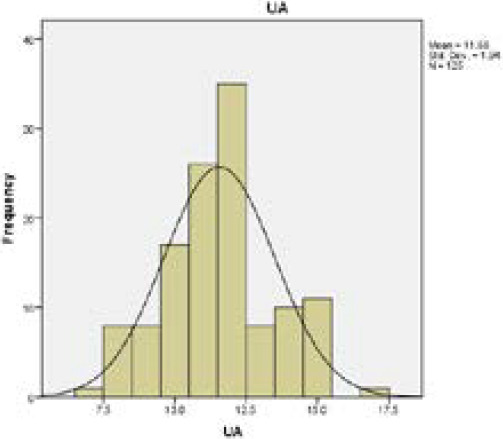
Histogram showing Normal Distribution for Upper Pharyngeal Airway Dimension.

Mean and standard deviation of upper pharyngeal airway is given (Table 1). Upper airway dimension was. The frequency of quantitative data of five different variables of airway, tongue dimension and hyoid bone position showed frequency distribution graph (Figure 1) with normal distribution approximately.

**Table 1:** Variations in Upper Pharyngeal Airway Dimension.

	Upper Pharyngeal Airway Dimension (Mean±S.D) (mm)
Female	11.68±1.96
Male	11.40±1.84
**Total**	**11.5±1.94**

**Table 2:** Parameters Related to Upper Pharyngeal Airway Dimension.

Abbreviations	Total (Mean±S.D) (mm)	Male (Mean±S.D) (mm)	Female (Mean±S.D) (mm)
Lower Pharyngeal Width	10.27±2.71	11.23±2.45	9.38±2.65
Tongue Height	34.73±2.84	35.82±2.95	33.72±2.34
Tongue Length	72.50±5.02	74.62±5.10	70.54±4.10
Hyoid Bone position	9.99±4.42	10.93±4.14	9.12±4.53

Mean age of participants involved in study, both males and females (Table 3).

**Table 3:** Demographic Data.

Age	Sex	n	Mean±S.D (yrs)
	Male	60	20.75±2.67
	Female	65	20.05±2.25
	Total	125	20.38±2.47

## DISCUSSION

Findings of our study, the mean upper pharyngeal airway dimension was 11.56±1.94mm, lower airway dimension was 10.27±2.71 mm, tongue height was 34.73±2.84mm, tongue length was 72.50±5.02mm and the position of hyoid bone was 9.99±4.42mm.

The aim of orthodontic treatment is to achieve maximum functional efficiency beside esthetic harmony. Like mastication, breathing is also one of the crucial functions performed by the craniofacial structure. Narrow airway passage leads to altered breathing and abnormal growth of functional components resulting abnormal morphologic pattern. Hence this study was performed with the objective of measuring airway dimension and to know the measure and the position of oropharyngeal structures, the tongue and hyoid bone. Therefore, while achieving esthetical harmony and masticatory functional efficiency, breathing has to be given equal importance.

Normal functional components will have normal functioning and normal morphological pattern of airway and tongue and hyoid bone position.^7^ Mouth breathing habit which has high correlation with malocclusion due to lowered positioning of the tongue, impairing facial morphology during growth were excluded from this study.

The ages of the sample of the study taken were 1827 years, where the lymphoid tissue growth would have regressed and adult size of the hard and soft tissues would have attained. Airway dimensions are established in early childhood, beside airway becomes naturally less obstructive with increasing age with gradual natural compensation.^8,9^

Lateral cephalograms in this study revealed smooth outline of the posterior pharyngeal wall without tonsillar hypertrophy and forward posturing of mandible associated with wider lower pharyngeal airway width.^10^

Descriptive statistic of five variables for gender revealed mean upper airway dimension as 11.40±1.84mm and 11.68±1.96mm, lower airway dimension as 11.23 ± 2.45mm and 9.38±2.65mm, tongue height as 35.82±2.95mm and 33.72±2.34mm, tongue length as 74.62±5.10mm and 70.54±4.10mm, the position of hyoid bone as 10.93±4.14mm and 9.12±4.53mm respectively for male and female.

In contrast to the study by Allhaija ESA and Al-khateeb SN,^1^ this study revealed tongue size thicker and longer for male than females, lower airway dimension also larger than females. Greater dimension of the parameters in our study could be due to greater craniofacial dimension in males than females. However, hyoid bone was lowerly positioned in males than females similar to our study. Disproportionate increase in tongue mass relative to oral cavity may cause downward movement of tongue as one grows which plays a role in anterior and inferior movement of hyoid bone especially in males.^11^ Similar airway dimension in males and females has also been observed in study by Mislik B, et al.^9^ in spite of differences of craniofacial dimension in males and females.

According to McNamara upper pharyngeal width is 15-20mm and lower pharyngeal width is 11 - 14 mm, being greater than the observed value in our study. Ethnic diversity resulting in greater craniofacial structure could be the reason, since the sample population in McNamara is Caucasian.^12^

For generalisability pharyngeal airway dimension can be used as reference value to know airway patency and a width of two mm or less in this region may indicate airway impairment. Greater width of lower pharyngeal width may suggest habitual anterior positioning of the tongue or tonsillar enlargement.^12^ Regarding hyoid bone greater distance of hyoid bone from mandibular plane may be associated with sleep apnea. Recently it is considered as compensatory response rather than predisposing factor for airway obstruction.^13^

This study is the two dimensional study of three dimensional glosso-pharyngeal structure in lateral cephalogram where they are proven to be reproducible.^5^ Hence it is still widely used and further studies may be undertaken since it is routinely taken for orthodontic diagnosis. Beside, though this parameter has been put forward by McNamara in the past years, recently disordered breathing like sleep disordered breathing has reintroduced this parameter.^12^

## CONCLUSIONS

Gender diversity regarding upper pharyngeal airway dimesnion was not observed in this study while other studies also revealed similarities in upper pharyngeal airway dimension between male and female, inspite of larger craniofacial dimension in males than females.

Lateral cephalograms taken routinely for orthodontic diagnosis could have additional parameter to evaluate airway dimension. We recommend further study of these parameters in hyperdivergent group with normal skeletal and dental relation.
